# Donor Variability Alters the Characteristics of Human Brain Microvascular Endothelial Cells

**DOI:** 10.3390/cimb47020073

**Published:** 2025-01-23

**Authors:** Jingyuan Ya, Ulvi Bayraktutan

**Affiliations:** 1 Stroke, Mental Health and Clinical Neurosciences, School of Medicine, University of Nottingham, Nottingham NG7 2UH, UK; 2 School of Medicine, Ankara Medipol University, Hacı Bayram Mah, Talatpaşa Blv No. 4, 06050 Altindag, Türkiye

**Keywords:** primary cell, endothelial cell, donor difference, blood–brain barrier, senescence

## Abstract

Primary brain microvascular endothelial cells (BMECs) are widely used in a large number of in vitro studies each year to better mimic their physiological characteristics in vivo. However, potential changes in primary endothelial cells stemming from donor variability or culture conditions may affect the reliability and reproducibility of the experiments. While working on a project regarding BMEC senescence, we noticed behavioral differences between two different batches of cells. Comparative analyses of cellular characteristics revealed that while one batch of BMECs developed a typical cobblestone morphology, the other batch displayed a spindle-shape morphology. Despite showing similar tubulogenic and barrier-forming capacities, the spindle-shaped BMECs displayed greater proliferation rates, stronger staining for CD34, a marker of stemness and higher resistance to oxidative stress-induced senescence and replicative senescence. Conversely, the spindle-shaped cells demonstrated a much weaker staining for the endothelial marker CD31. Taken together, these findings indicate that it is important to scrutinize endothelial characteristics to ensure experimental accuracy when cellular responses markedly vary between the so-called endothelial cells.

## 1. Introduction

Endothelial cells cover the inner surface of all blood vessels and synthesize a large number of substances in response to several chemical (e.g., acetylcholine and calcium ionophore), physical (flow and shear stress) and humoral (autacoids and bradykinin) stimuli to regulate vascular tone, coagulation, angiogenesis, inflammation and vascular permeability [1,2]. Angiotensin II, endothelin-1, prostacyclin, endothelium-derived hyperpolarizing factor and nitric oxide constitute some of the key substances released by the endothelial cells. In addition to maintaining vascular homeostasis, endothelial cells also help form specific barriers in certain organs to control vascular permeability. The blood–brain barrier (BBB) in the central nervous system is one such barrier. The BBB regulates the selective passage of elements between the blood and the brain parenchyma. Although capillary basement membrane and astrocyte end-feet represent other key components of the BBB, the restraining role of the BBB is largely realized by the continuous presence of inter-endothelial cell tight junctions that are formed by several transmembrane and associated cytoplasmic proteins, notably occludin and zonula occludens-1 (ZO-1) [3]. Bearing this in mind, it is reasonable to hypothesize that any change in endothelial phenotype and/or characteristics can profoundly influence the expression and localization of these proteins and therefore adversely affect the integrity and function of the BBB [4].

To better represent the organ-specific characteristics in vitro, primary endothelial cells are commonly used in experimental settings. These cells are obtained directly from the tissue and are not modified in any way [5]. Hence, they display physiological characteristics similar to the in vivo state. This not only renders studies concerning normal cellular physiology and biochemistry possible but also enables the detection of cellular responses to a large number of therapeutics and physio-pathological phenomena such as senescence, oxidative stress and hyperglycemia [6,7,8]. However, the variability observed in primary endothelial cells obtained from different donors or induced by different subculture practices is a major issue for endothelial cell biologists [9]. Hence, it may be advisable to pre-screen endothelial cells for their phenotypic and functional characteristics before performing the planned experiments in order not to waste valuable resources and ensure scientific rigor.

In light of the above, this study investigated the characteristics of two different batches of brain microvascular endothelial cells (BMECs) using a variety of morphological, biochemical and functional studies.

## 2. Materials and Methods

### 2.1. Cell Culture

Two vials of human brain microvascular endothelial cells (HBMECs) and a single vial of human brain microvascular pericytes (HPs) and human brain astrocytes (HAs) were purchased at passage 3 from different commercial companies. All cells were cultured at 37 °C in their respective complete media in a humidified atmosphere of 75% N_2_, 20% O_2_ and 5% CO_2_. Cellular morphology was visualized and photographed with the Digipad connected to a light microscope (Leica DFC3000 G, Wetzlar, Germany) under 10× magnification.

HBMECs were exposed to 400 μM of H_2_O_2_ for 48 h and incubated in normal complete media for 12 days to develop stress-induced premature senescence (SIPS).

### 2.2. Immunocytochemistry

HBMECs grown on glass coverslips were subjected to different experimental conditions before fixing (in 4% paraformaldehyde) and permeabilizing (in 0.1% Triton X-100) for 15 min at room temperature. After blocking with 1% bovine serum albumin in PBST (0.1% Tween 20 in PBS) for 30 min at room temperature, the cells were incubated overnight at 4 °C with primary antibodies, including ZO-1 (33-9100, Thermofisher, Waltham, MA, USA), CD31 (3528, Cell Signaling Technology, Danvers, MA, USA), CD34 (ab81289, Abcam, Cambridge, UK), α-SMA (ab7817, Abcam) and phosphor-Histone H2AX (Ser139, 9718, Cell Signaling Technology). On the following day, the cells were washed and incubated with FITC-labelled or Texas Red-labelled secondary antibodies (ab6785, ab6717, ab6719, Abcam) for 1 h at room temperature in the dark.

To visualize F-actin microfilaments, the cells were blocked as above and incubated with the DyLight 594 Phalloidin (12877, Cell Signaling Technology) for 15 min. To stain nuclei, the cells were incubated with 4,6-diamidino-2-phenylindole (DAPI) for 3 min after washing. The coverslips were then mounted onto glass slides using mounting medium (Vector Laboratories, Peterborough, UK). The cells were photographed under 40× magnification by a fluorescence microscopy (Leica DFC3000 G).

### 2.3. Western Blotting

After subjecting them to different experimental conditions, the cells were washed with ice-cold PBS and scraped in 1× RIPA buffer (lysis buffer) containing 10 μL/mL protease inhibitor cocktail and 1 mM sodium orthovanadate. The supernatant was collected from the cell lysate by centrifugation at 14,000× *g* (4 °C) for 15 min. Protein concentrations were detected using the BCA protein assay kit (23227, Thermofisher, Waltham, MA, USA).

Total protein samples (20 μg) were mixed with 4% lithium dodecyl sulfate (4 × LDS) sample buffer (MPSB, Sigma, Burlington, MA, USA) in a ratio of 3:1 and heated at 75 °C for 5 min to open the tertiary/quaternary structures. The protein sample mix was then run on 8% SDS-polyacrylamide gels at 120 V in running buffer containing 25 mM Tris-HCl pH 8.3, 190 mM glycine, 0.1% (*w*/*v*) SDS. After electrophoresis, the protein samples were transferred to a PVDF membrane in cold transfer buffer (25 mM Tris-HCl pH 8.3, 190 mM glycine, 20% (*v*/*v*) methanol). The membrane was blocked with 5% bovine serum albumin (BSA) in washing buffer (20 mM Tris-HCl pH 7.5, 150 mM NaCl, 0.1% (*v*/*v*) Tween-20) at room temperature for 1 h on a rocker at 30 oscillations/minute before incubating overnight with anti-CD31 (3528, Cell Signaling Technology), anti-ZO-1 (40-2200, ThermoFisher) and anti-β-actin (ab8227, Abcam) in 2% BSA in washing buffer on a tube roller at 4 °C. β-actin was used as an internal loading control. The membranes were then washed 5 times (5 min each time) with washing buffer on a rocker at 70 oscillations/minute. Membranes were then incubated with IRDye-labeled (800CW/680CW) secondary antibodies (1:10,000 diluted with 2% BSA in washing buffer) for 1 h at room temperature in a dark box on a rocker (30 oscillations/minute). After repeating the washing step mentioned above, the membranes were scanned and the protein bands were detected using the Odyssey Fc System before analyzing their intensities via Image Studio software (5.0, Li-Cor Biotechnology, Lincoln, NE, USA).

### 2.4. Assessment of In Vitro Blood–Brain Barrier Integrity and Function

A triple cell culture model of human BBB was set up as before [10]. For this, ~1 × 10^5^ HAs were first seeded on the basolateral side of the transwell insert (polyester membrane, 12 mm diameter, 0.4 μm pore size, Corning, New York, NY, USA) and incubated for 4–6 h. Once the cells adhered to the membrane, the inserts were inverted to the original orientation and cultured in sterile 12-well plates containing fresh astrocyte media to about 90% confluence. HBMECs (5 × 10^4^) were then seeded onto the apical side of the inserts containing HAs and cultured to full confluence. The culture media in luminal and abluminal compartments were changed daily with fresh endothelial cells and astrocyte complete media to nourish the cells until they reached full confluence. In the meantime, HPs (15 × 10^4^ cells) were seeded into a new 12-well plate and cultured in pericyte complete medium to full confluence. The inserts containing fully confluent HBMECs and HAs were transferred to the well plate containing confluent HPs, to establish the triple culture model of human BBB.

To assess the integrity of the BBB, transendothelial electrical resistance (TEER) was measured using the EVOM manual meter and STX electrodes (World Precision Instruments, Hertfordshire, UK). For this, two electrodes with unequal lengths were placed vertically into the luminal and abluminal chambers without touching the sidewall. The TEER value for each insert was calculated as the average of three different readings obtained from three different positions.

To assess the permeability of the BBB, paracellular flux of sodium fluorescein (NaF, 376 Da) was studied in that the inserts were rinsed with warm Hank’s Balanced Salt Solution (HBSS, Sigma, St. Louis, MI, USA) and transferred into fresh 12-well plates containing 2 mL of HBSS. A total of 500 μL of 50 μg/mL NaF was then added into the luminal chamber of each insert and the plates with the inserts were incubated for 1 h at 37 °C in a CO_2_ incubator. After incubation, 400 μL of samples was collected from the luminal and abluminal chambers and 100 μL of each sample was pipetted into 96-well plates in triplicate. The average concentration of dye in each chamber was calculated based on the fluorescence for NaF assay (excitation 485 nm and emission 520 nm) of the samples with the FLOUstar Omega plate reader (BMG Labtech Ltd., Aylesbury, UK). The paracellular flux of the dye was calculated using the following formulae: Cleared volume (μL) = Abluminal reading × 500/luminal reading.

### 2.5. Tube Formation Assay

Level of tubulogenesis on growth factor reduced Matrigel (Corning, New York, NY, USA) was studied to evaluate the angiogenic capacity of the HBMECs. For this, 200 μL pipette tips and cooling core were chilled in a −20 °C freezer for at least 24 h before the start of experiments. Matrigel was thawed at 4 °C overnight and pipetted into 96-well plates (50 μL per well) using pre-chilled tips on a cooling core. After carefully checking and removing the bubbles, the well plates were incubated at 37 °C for 1 h to facilitate the solidification of Matrigel. Meanwhile, HBMECs were detached with trypsin and re-suspended in cell culture medium. After counting, HBMECs (8 × 10^3^ cell/150 μL culture medium) were seeded in 96-well plates pre-coated with Matrigel and incubated at 37 °C for 4 h. The formation of the tubular structures was visualized and photographed using the Digipad connected to a light microscope (Leica DFC3000 G, Wetzlar, Germany). Using Angiogenesis Analyzer plugin ImageJ software (version 1.52k, NIH, Bethesda, MD, USA), the characteristics of the tubule networks, i.e., total number and total length of segments, were analyzed.

### 2.6. Senescence-Associated β-Galactosidase Activity

HBMECs were seeded in 12-well plates and cultured to around 70% confluence in complete cell culture medium. The SA-β-galactosidase activity was measured as an index of senescence using a β-galactosidase staining kit (APExBIO, Houston, TX, USA). In brief, cells were washed with warm PBS and fixed with the fixation solution at room temperature for 10 min, then washed with PBS and incubated in staining solution mix at 37 °C overnight. The cells were visualized and photographed using a light microscope (Leica, DFC3000 G, Wetzlar, Germany) under 20× magnification. Cells with blue dye were regarded as senescent. The numbers of β-gal-positive cells and total cells were counted manually in four randomly chosen areas of each well before working out the percentage of SA-β-gal-positive cells. The average of four areas was used in statistical analyses. Each experiment was repeated at least three times using three different biological replicates.

### 2.7. Statistical Analyses

The data are displayed as the mean value ± standard error of the mean (SEM) from at least three independent experiments. Variations across different groups were determined using an unpaired *t*-test. *p* < 0.05 was considered statistically significant.

## 3. Results

### 3.1. HBMECs Display Different Phenotypes

**Figure 1:**
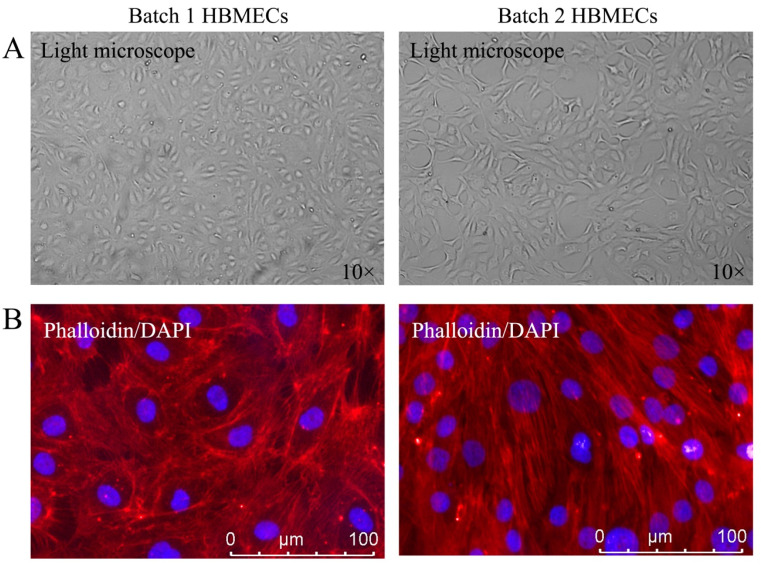
Comparison of human brain microvascular endothelial cell (HBMEC) morphology. While the first batch of HBMECs displayed typical cobblestone morphology, the second batch of HBMECs possessed somewhat elongated and spindle-shaped morphology (**A**). Examination of the cytoskeletal organization by actin microfilament staining confirmed these characteristics in respective cells (**B**).

To investigate whether HBMECs purchased from different commercial companies might have phenotypic differences, the cells were first visualized using a light microscope. As illustrated in Figure 1A, while one set of cells displayed a typical cobblestone morphology associated with confluent endothelial cells (batch 1), the other set exhibited a bigger and more elongated cellular morphology (batch 2). Further scrutiny of cellular appearance with actin microfilament staining confirmed that cells from batch 2 developed bigger, elongated and spindle-shaped morphology. Furthermore, while the cells with cobblestone morphology established a visible monolayer, spindle-shaped cells produced a multilayered appearance (Figure 1B).

### 3.2. HBMECs Express Endothelial Markers Differently

**Figure 2:**
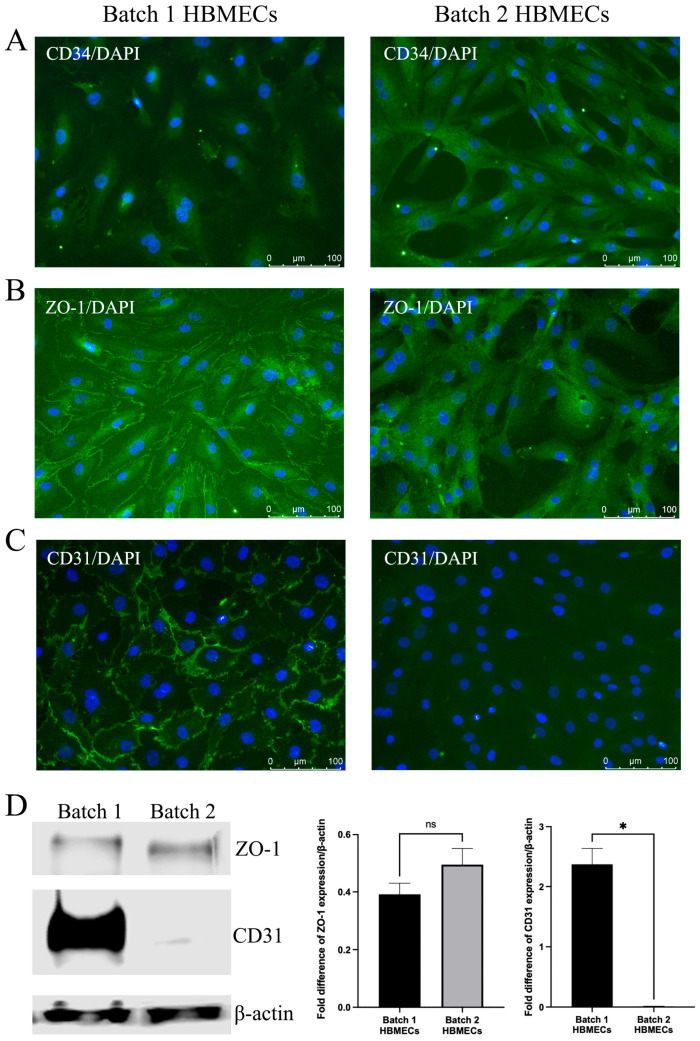
Markers for endothelial stemness and maturity are distinctly expressed in human brain microvascular endothelial cells (HBMECs). Compared to cells with cobblestone morphology, HBMECs with elongated morphology had strong CD34 (**A**) and ZO-1 (**B**) staining in cytoplasm. However, they stained negative for CD31 (**C**) and possessed markedly lesser quantity of its protein (**D**). For Western analyses, data are expressed as mean ± SEM from two separate experiments. * *p* < 0.05 compared to batch 1, ns = not significant compared to batch 1.

Contrary to HBMECs with cobblestone morphology, HBMECs with elongated morphology displayed strong CD34 staining in the cytoplasm. CD34 is regarded as an important marker of hematopoietic stem/progenitor cells and vascular endothelial progenitor cells (Figure 2A). In contrast, HBMECs with cobblestone morphology manifested a typical ZO-1 plasma membrane staining, which appeared to localize to the cytoplasm in elongated HBMECs (Figure 2B). Similar to ZO-1, batch 1 HBMECs also showed a prominent staining of CD31 on plasma membrane. However, no CD31 staining could be detected in batch 2 HBMECs (Figure 2C). Western analyses of total protein samples obtained from batch 1 and batch 2 cells substantiated the significantly depleted presence of CD31 in batch 2 cells and ZO-1 level remained similar between batches (Figure 2D).

### 3.3. Scrutiny of Alpha-Smooth Muscle Actin Expression in Endothelial Cells

**Figure 3:**
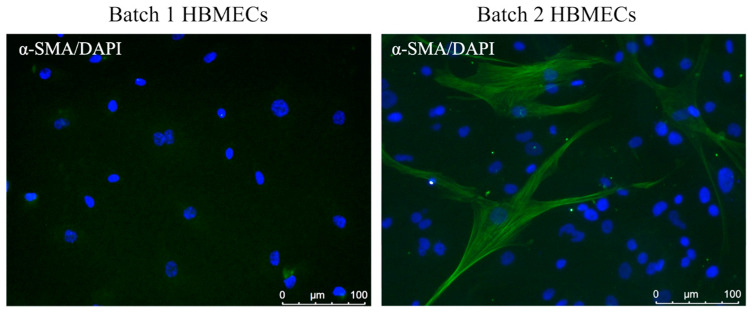
Expression of alpha-smooth muscle actin (α-SMA), a marker for fibrogenic cells in human brain microvascular endothelial cells (HBMECs). Despite absence of α-SMA in cobblestone-shaped cells, few elongated HBMECs were stained positive for the same marker.

Although HBMECs with cobblestone morphology were negative for alpha-smooth muscle actin (α-SMA), few cells with elongated morphology displayed positive staining for α-SMA (Figure 3), a marker for pericytes and myofibroblastic cells [11].

### 3.4. Effect of Changes in Endothelial Cell Characteristics on Functional Activity

**Figure 4:**
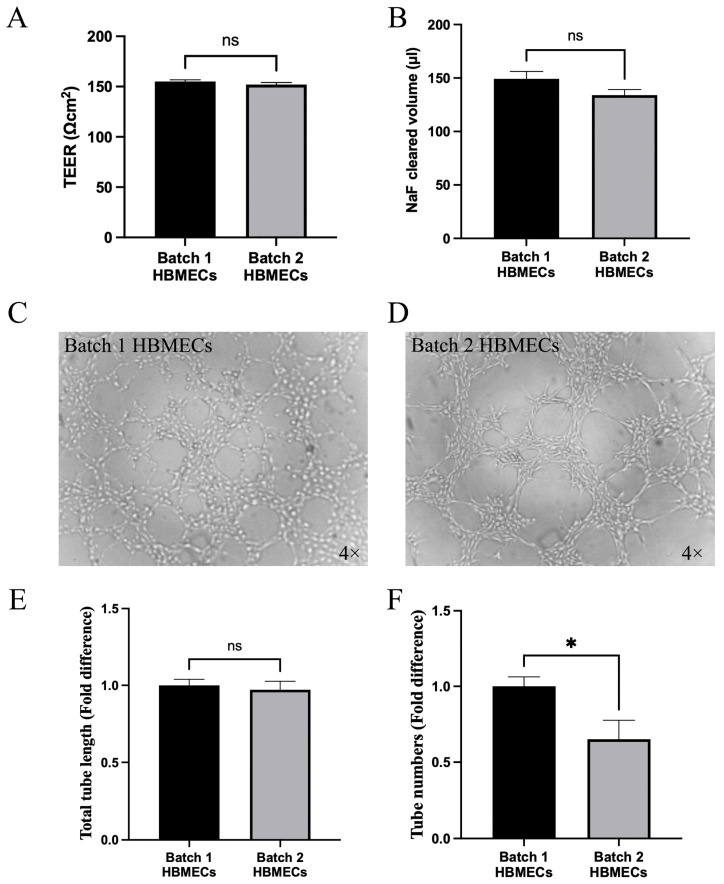
Human brain microvascular cells (HBMECs) with different morphology and characteristics formed equally tight and functional blood–brain barrier in the presence of human astrocytes and pericytes. These were supported by similar TEER readings (**A**) and a similar level of paracellular flux of sodium fluorescein (**B**). HBMECs with cobblestone morphology formed more circular (**C**,**D**) and greater numbers (**E**,**F**) of tubules on Matrigel than elongated counterparts. Data are expressed as mean ± SEM from three independent experiments. * *p* < 0.05 compared to batch 1, ns = not significant compared to batch 1.

As HBMECs constitute the most important cellular component of the BBB, the capacity of different batches of HBMEC to form the BBB was evaluated. The results showed that when combined with HA and HP, both cobblestone-shaped and elongated HBMECs formed an equally tight and functional BBB, as evidenced by similar TEER readings and NaF flux (Figure 4A,B). Furthermore, both sets of cells established coherent tubule networks on Matrigel, proving their capacity for angiogenesis in vivo (Figure 4C,D). Even so, tubules formed with batch 2 HBMECs appeared to be less orbicular and fewer in number (Figure 4E,F).

### 3.5. Endothelial Cells Respond to Senescence-Inducing Stimuli Differently

**Figure 5:**
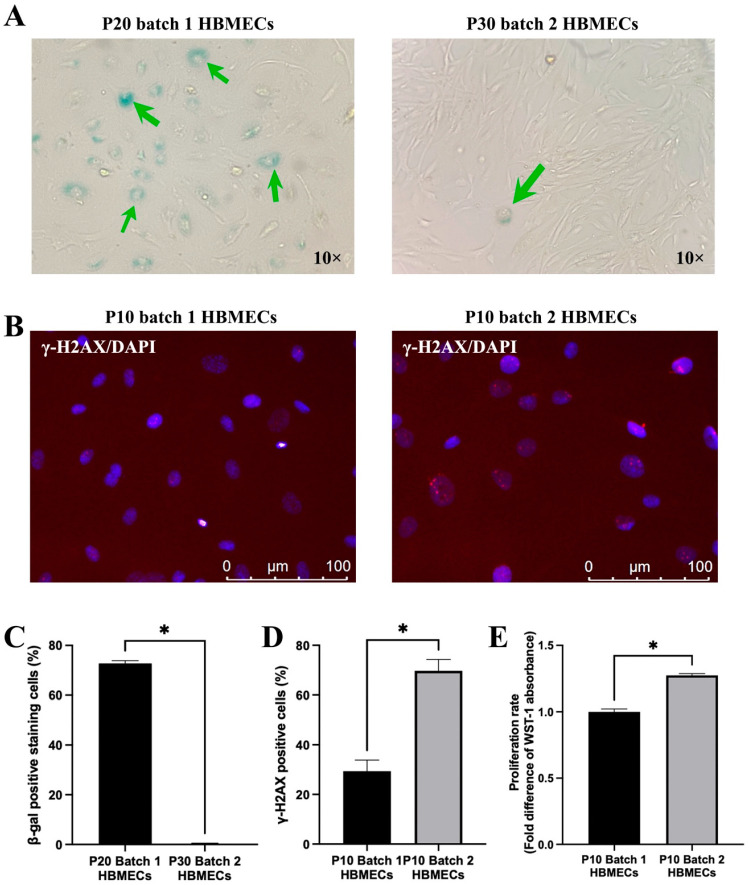
Human brain microvascular endothelial cells (HBMECs) with cobblestone morphology stained positive for SA-β-gal at earlier passages as indicated by green arrows (**A**,**C**). They also showed relatively lower DNA damage at earlier passages (**B**,**D**). The same cells had lower proliferation rates compared to those with elongated morphology (**E**). Data are expressed as mean ± SEM from three separate experiments. * *p* < 0.05 compared to batch 1.

HBMECs with elongated morphology displayed a higher proliferation rate and greater resistance to replicative senescence, a laboratory model of chronological aging mimicked by repetitive passaging, compared to the cells with cobblestone morphology. Indeed, while these cells continued to be highly proliferative even at passage 30, cells from batch 1 ceased to proliferate at passage 20, where at least 70% of the cells stained positive for SA-β-gal (Figure 5A,C). Intriguingly, HBMECs with elongated morphology generated higher levels of DNA damage foci at a relatively early passage with no sign of cell cycle arrest, implying a faster DNA repair rate (Figure 5B,D), as ascertained by significantly higher proliferation rate at this passage (Figure 5E).

**Figure 6:**
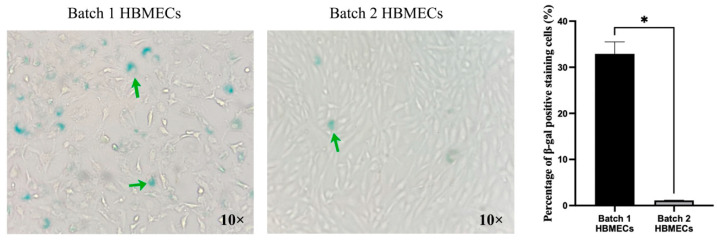
Human brain microvascular endothelial cells (HBMECs) with elongated morphology better resist H_2_O_2_-induced premature senescence. Green arrows indicate cells stained positive for SA-β-gal. Data are expressed as mean ± SEM from three independent experiments. * *p* < 0.05 compared to batch 1.

Similar to replicative senescence, premature senescence induced by exogenous stress, i.e., oxidative stress, realized by exposure to a higher level of H_2_O_2_ (400 μM), also evokes higher rates of senescence in HBMECs with cobblestone morphology compared to elongated cells (Figure 6).

## 4. Discussion

Primary cells are obtained directly from an organ of interest without modification and therefore better represent the characteristics of this particular organ in laboratory settings compared to any established cell line. However, the phenotype and behavior of primary cells may vary from donor to donor or even between different collections from the same donor [12]. It is possible that differences in donors’ age, sex, genetics and health status may play a key role in these differences [13,14]. Primary cells proliferate a finite number of times in laboratory conditions before senescing. Bearing these in mind while working on a project probing the molecular mechanisms involved in HBMEC senescence, we noticed that two different batches of HBMECs responded to the repetitive passaging differently and acquired senescence phenotype at different time points (passage 20 vs. passage > 30), as ascertained by positive staining with SA-β-gal. Subsequently, differences in responding to oxidative stress-induced senescence were also observed between different batches of HBMECs. To provide an explanation for these differences, the current study comparatively assessed the morphological and functional features of cells purchased. These, alongside DNA/RNA sequencing and scrutiny of cell-specific markers by immunofluorescence or flow cytometry, make up the common methodologies to identity a particular cell type [5,15].

Cellular morphology is an outcome of several cellular processes and can yield some useful information about the properties of a certain type of cell [16]. Visual inspection of endothelial cells used in our experiments by light microscopy revealed that while one batch of HBMECs (batch 1) developed a typical cobblestone morphology, cells from the other batch took on more of an elongated spindle-shaped morphology. This morphological diversity of endothelial cells suggests the remodelling of the cytoskeleton in response to a variety of physio(patho)logical stimuli such as shear stress or inflammation [17,18,19]. Cobblestone morphology indicates a mature endothelial cell line with normal functions [20], while spindle-shape morphology indicates elevated rates of proliferation, migration and genomic instability [21,22]. Staining of actin microfilaments in the same cells confirmed the morphological differences and pinpointed possible differences in cellular characteristics and function. In addition to providing a structural framework for the cell, the actin cytoskeleton also plays pivotal roles in cell shape, division and migration and, depending upon cellular demand, constantly undergoes polymerization and depolymerization [23]. Uniquely in endothelial cells, actin helps form intercellular junctions that help maintain the blood–tissue barrier, notably the BBB in the CNS [24]. Observation of similar TEER and NaF flux readings in BBB established with human astrocytes, pericytes and either HBMEC type indicated that differences in actin cytoskeletal organization did not affect barrier integrity or function. Preservation of the BBB integrity and function indicates adequate availability and appropriate localization of major tight junction and associated proteins between adjacent endothelial cells. The functional and dynamic complexes established by these proteins effectively seal the inter-endothelial cellular space and prevent paracellular leakage of solutes, ions and water [25]. Intriguingly in this study, ZO-1 has almost completely disappeared from the plasma membrane of elongated HBMECs and appeared exclusively to localize to the cytoplasm with no adverse effect on BBB integrity or function. Further characterization of cells with endothelial cell-specific marker CD31 [26] revealed that elongated HBMECs were negative for CD31, implying a phenotype of incomplete endothelial differentiation or maturation [27]. Under normal circumstances, CD31 regulates a variety of crucial functions in endothelial cells, including barrier formation, angiogenesis, anti-apoptosis and immune modulation [28]. Endothelial cells with low CD31 expression have been shown to exhibit increased proliferative potential and enhanced resistance to oxidative stress compared to cells with high CD31 expression [29]. In support of these results, spindle-shaped cells with low CD31 expression demonstrated resistance to oxidative stress-induced senescence and a high proliferation rate compared to CD31-positive cells in this study. Significantly greater staining for CD34 in spindle-shaped cells corroborates the lack of endothelial differentiation and strongly supports endothelial immaturity. It is of note here that CD34 is primarily employed as a biomarker for hematopoietic stem/progenitor cells. However, it can also identify several non-hematopoietic cells, such as endothelial progenitor cells that are released by bone marrow in response to a vascular injury but also mediate blood vessel formation during development [30]. Endothelial-to-mesenchymal transition (EndoMT) is a transdifferentiation process triggered by various signals and is characterized by a progressive loss of endothelial-specific markers and the acquisition of mesenchymal phenotype [31]. Observation of CD31 downregulation alongside a concomitant overexpression of CD34 in the second batch of HBMECs suggests a possible EndoMT in these cells, a hypothesis supported by the positive staining of cells for α-SMA.

The ability of both batches of cells to form tubules on Matrigel indicates their capacity to induce angiogenesis and suggests the possession of endothelial properties by both cells. Given that endothelial progenitor cells can also form tubules on Matrigel, these findings suggest that the elongated HBMECs may indeed be some sort of immature endothelial cell [32]. Even though these cells formed less orbicular and fewer tubules compared to batch 1 HBMECs, it was unlikely that they were early endothelial progenitor cells, as they established tubular structures on Matrigel [33]. The elongated HBMECs displayed greater resistance to senescence while intriguingly expressing higher levels of γH2AX, a DNA damage marker. Apart from indicating senescence, high expression levels of γH2AX may also signify an increase in DNA repair-related genes such as p53 and an increase in proliferative potential [34].

The findings of this study highlight the importance of pre-screening primary cells during the early phase of a planned study, especially when cells from different sources are to be employed. In terms of endothelial cells, a panel of specific markers and assays can be utilized to assess both endothelial cell characteristics (CD31, CD34, and von Willebrand factor) and functionality (tubulogenesis and angiogenesis). These will ensure the true nature of cells and produce reliable results.

There are some limitations to this study. Firstly, investigation of the expression and localization of other major tight junction proteins, namely occludin and claudin-5 in both HBMEC populations, would have provided some additional information on experiments concerning the tightness and function of the BBB. Secondly, the application of specific markers for fibroblasts and epithelial cells to determine cellular identity would have been useful to prove the endothelial nature of both cells and dismiss contamination with other cells. Thirdly, genomic analysis could have provided a more comprehensive explanation as to the morphological and behavioral variations observed between the two different batches of endothelial cells.

## 5. Conclusions

In conclusion, primary endothelial cells from different collections may behave differently and lead to the generation of irreproducible and possibly flawed results. It is therefore highly advisable to explore the endothelial characteristics of the cells used in a given study if the cellular behaviors do not match.

## Data Availability

Data are available on reasonable request from the authors.
